# Differential Ligand Binding to a Human Cytomegalovirus Chemokine Receptor Determines Cell Type–Specific Motility

**DOI:** 10.1371/journal.ppat.1000304

**Published:** 2009-02-20

**Authors:** Jennifer Vomaske, Ryan M. Melnychuk, Patricia P. Smith, Joshua Powell, Laurel Hall, Victor DeFilippis, Klaus Früh, Martine Smit, David D. Schlaepfer, Jay A. Nelson, Daniel N. Streblow

**Affiliations:** 1 Department of Molecular Microbiology and Immunology and The Vaccine and Gene Therapy Institute, Oregon Health & Science University, Portland, Oregon, United States of America; 2 Department of Immunology, The Scripps Research Institute, La Jolla, California, United States of America; 3 Leiden/Amsterdam Center for Drug Research, Division of Medicinal Chemistry, Faculty of Chemistry, Amsterdam, The Netherlands; Harvard Medical School, United States of America

## Abstract

While most chemokine receptors fail to cross the chemokine class boundary with respect to the ligands that they bind, the human cytomegalovirus (HCMV)-encoded chemokine receptor US28 binds multiple CC-chemokines and the CX_3_C-chemokine Fractalkine. US28 binding to CC-chemokines is both necessary and sufficient to induce vascular smooth muscle cell (SMC) migration in response to HCMV infection. However, the function of Fractalkine binding to US28 is unknown. In this report, we demonstrate that Fractalkine binding to US28 not only induces migration of macrophages but also acts to inhibit RANTES-mediated SMC migration. Similarly, RANTES inhibits Fractalkine-mediated US28 migration in macrophages. While US28 binding of both RANTES and Fractalkine activate FAK and ERK-1/2, RANTES signals through Gα12 and Fractalkine through Gαq. These findings represent the first example of differential chemotactic signaling via a multiple chemokine family binding receptor that results in migration of two different cell types. Additionally, the demonstration that US28-mediated chemotaxis is both ligand-specific and cell type–specific has important implications in the role of US28 in HCMV pathogenesis.

## Author Summary

Chemokines are small cytokines that are critical for recruiting and activating the cells of the immune system during viral infections. A number of viruses, including the large herpes virus human cytomegalovirus (HCMV), encode mechanisms to impede the effects of chemokines or have gained the ability to use these molecules to their own advantage. HCMV encodes multiple chemokine receptors including US28, which binds two different classes of chemokines namely the CC and CX_3_C families. In this report, we demonstrate that US28 binding to a CC chemokine elicits different responses compared to when binding to Fractalkine, the only CX_3_C chemokine. RANTES (CC chemokine) binding to US28 mediates smooth muscle cell migration, but Fractalkine blocks this process in a dose-dependent manner. However, Fractalkine binding to US28 can specifically mediate the migration of macrophages, another important cell type during viral pathogenesis. We explored the intracellular signaling pathways responsible for each migration event and determined that they differ in the G-proteins that are coupled to US28 following addition of ligand and that this occurs in a cell type–specific manner. These results provide a new mechanism for HCMV acceleration of vascular disease via the specific migration of macrophages and provide the first example of cell type–specific migration via multiple chemokines binding to a single receptor.

## Introduction

All β and γ-herpesviruses encode molecules with the potential to modulate the host immune response, including chemokines and/or chemokine receptor homologs. The β-herpesvirus human cytomegalovirus (HCMV) encodes a CXC-chemokine (UL146), a potential CC-chemokine (UL128), and four potential chemokine receptors (US27, US28, UL33 and UL78) with the most characterized being US28 [1]–[4]. Chemokines are small, inducible cytokines that have critical roles in the induction and promotion of cellular migration and activation upon binding 7-transmembrane spanning G-protein coupled receptors (GPCRs). There are four major chemokine subfamilies that are categorized according to the spacing of the first two conserved amino-terminal cysteine residues: CC-, CXC-, CX_3_C- and XC-. Most chemokine receptors bind a limited subset of ligands belonging to a single subfamily. The ability to bind multiple ligands from different chemokine subfamilies is unique to a select few receptors including the Duffy antigen/receptor for chemokine (DARC-receptor) and the HHV-8-encoded chemokine receptor Orf74. These receptors have been reported to bind to both CC- and CXC-chemokines [5]–[7]. US28 also binds multiple ligands from different subfamilies. US28 contains homology to CC-chemokine receptors, with greatest homology to CCR1 [8] and binds to a broad spectrum of CC-chemokines with high affinity including: RANTES, MCP-1, MIP-1α and MIP-1β [9]. Interestingly, US28 also binds the CX_3_C-chemokine Fractalkine and with greater affinity than CC-chemokines. Although the N-terminal 22 amino acids of US28 have been shown to be required for binding of both chemokine classes [10], binding is not competed with saturating quantities of selected CC-chemokines [11]. Therefore, Fractalkine is predicted to bind unique regions of US28 compared to the CC-chemokines. Indeed, recent mutagenesis studies of the US28 N-terminus revealed that the phenylalanine residue at position 14 of US28 is important for binding of CC chemokines but is dispensable for Fractalkine binding, while mutation of tyrosine 16 negatively effects binding of both classes of chemokines [12].

Binding of chemokines to their respective receptors stimulates the cell type-dependent activation of a plethora of cellular signaling pathways specific to the chemokine/receptor pair. The CC-chemokines are known to be potent stimulators of cellular activation through US28. For example, in 293 cells, RANTES binding to US28 activates ERK-1/2 pathways through the G-proteins Gαi1 and Gα16 [13]. We have previously demonstrated that US28-mediated SMC migration is ligand-dependent requiring either exogenously added RANTES or endogenously expressed MCP-1 [14]. This migratory process is not blocked by treatment with pertussis toxin (PTX), a Gαi/o G-protein inhibitor, suggesting that other G-proteins are involved in this event [14]. Subsequent studies revealed that US28 couples with Gα12/13, promoting SMC migration and ligand-dependent signaling through the small G-protein RhoA [15]. US28 mediated SMC migration is also sensitive to treatment with protein tyrosine kinase (PTK) inhibitors, and the PTKs focal adhesion kinase (FAK) and Src are activated in US28 expressing cells upon RANTES binding [16]. Dominant negative inhibitory FAK molecules blocked US28 induced SMC migration suggesting that FAK activation is critical for US28 mediated SMC motility [16].

Although US28 binding to CC-chemokines leads to the activation of a multitude of cellular signaling pathways, the only activities associated with US28 binding to Fractalkine involve the modulation of constitutive signaling activity [17]–[19]. Treatment of US28 expressing cells with Fractalkine or the US28 synthetic inverse agonist VUF2274 leads to substantial decreases in the ability of US28 to promote the Gαq/11 dependent constitutive activation of phospholipase-C (PLC) and NF-κB, whereas MCP-1 and RANTES have only negligible effects on constitutive signaling levels [10],[18]. Additionally, Fractalkine treatment of US28 expressing HEK293A cells reduces constitutive US28 phosphorylation [19] and steady state levels of surface US28, but has little influence on the rapid endocytosis observed in HeLa cells [17]. The ability of US28 to efficiently bind ligands from multiple chemokine subfamilies coupled with the vastly different signaling responses elicited by divergent ligands is intriguing and suggests that US28 signaling is not only ligand and cell-type dependent, but also ligand-specific.

In the current study, we investigate the signaling potential of US28 upon stimulation with CC-chemokines compared to the CX_3_C-chemokine Fractalkine. We demonstrate that Fractalkine binding to US28 inhibits the ability of CC-chemokines to induce SMC migration. RANTES, MCP-1, and Fractalkine binding to US28 induced similar levels of FAK activation in fibroblasts. Overexpression studies indicate that RANTES-mediated stimulation of FAK occurs via a Gα12-dependent mechanism while Fractalkine utlilzes Gαq. In contrast to SMC, when US28 is expressed in macrophages, Fractalkine stimulation produces robust migration These results suggest that US28-signaling is ligand-specific and cell type-specific, and that RANTES and Fractalkine promote differential G-protein coupling leading to the activation of alternative signaling pathways depending on the cell-type and the complement of endogenously expressed G-proteins.

## Results

### Ligand-Specific US28 Mediated Smooth Muscle Cell Migration

**Figure 1:**
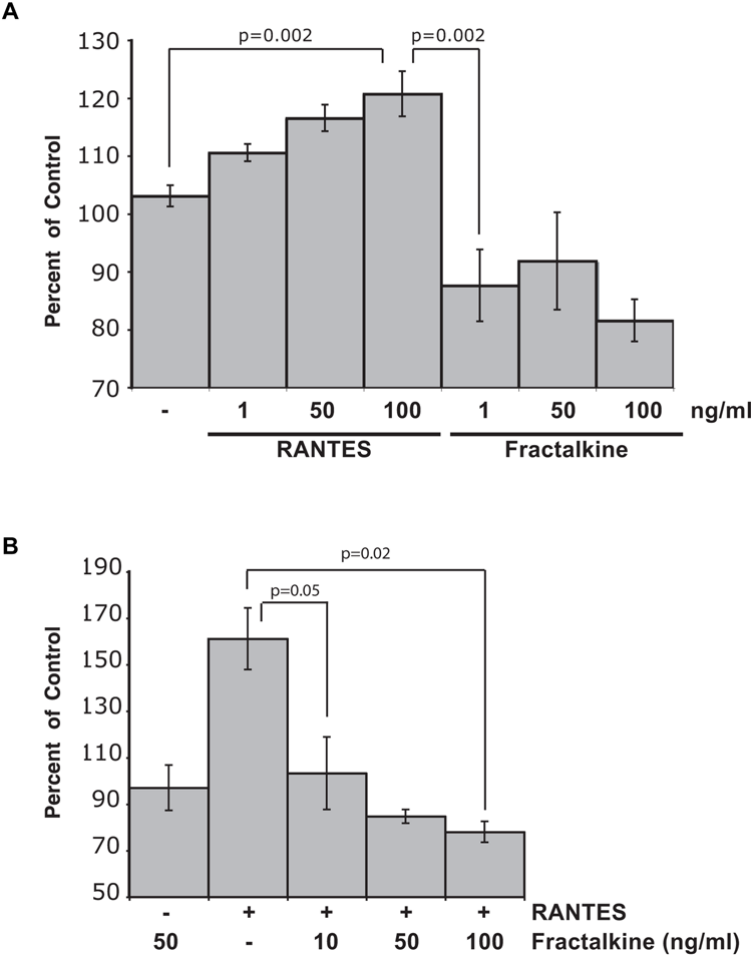
Fractalkine inhibits US28-mediated SMC migration induced by RANTES. (A) SMC migration assays were performed on cells infected with adenovirus expressing US28-HA treated with either RANTES or Fractalkine at the indicated concentrations. Data are represented as a percentage of unstimulated cells infected with control adenovirus transactivator only. For all conditions, n>6 from two independent experiments. (B) SMC migration assays were performed on US28-expressing cells treated with RANTES, Fractalkine or 40ng/ml of RANTES and the indicated concentrations of Fractalkine as a competing ligand.

The unique ability of US28 to bind both CC- and CX_3_C-chemokine ligands raises the question of whether US28 signaling is not only ligand-dependent, but also ligand-specific [9],[13],[20],[21]. To determine whether US28 signaling and SMC migration are ligand-specific, we performed SMC migration and signaling assays on US28 adenovirus expressing primary rat SMC in the presence of RANTES or Fractalkine. In this assay, RANTES readily induced US28-mediated SMC migration, however, increasing concentrations of Fractalkine failed to stimulate cellular motility above Ad-tet-transactivator (Trans) infected and RANTES stimulated controls, indicating that not all US28 ligands evoke the same functional response (Figure 1A). Visual analysis of the cells prior to and following the migration assay indicated that the lack of migration was not due to overt cell death mediated by US28 expression and subsequent treatment with Fractalkine (data not shown). A competition assay was performed to determine whether Fractalkine inhibits the ability of RANTES to induce SMC migration. In these experiments, RANTES alone promoted SMC migration, as expected. However, Fractalkine, at concentrations as low as 10ng/ml, was sufficient to block RANTES-mediated SMC migration (Figure 1B) suggesting that Fractalkine is a competitive inhibitor to CC-chemokine induced SMC migration.

Since RANTES but not Fractalkine caused the migration of US28 expressing SMC and since Fractalkine blocks this migration event, we hypothesized that the difference in the ability to promote motility occurred at the level of signaling. To determine whether there exists a gross difference in the ability of these chemokine receptors/ligands to modulate intracellular signaling cascades, host transcriptional profiles were examined using DNA microarrays. Interestingly, the cellular gene expression profile of US28-expressing SMC stimulated with RANTES substantially differs from the profile obtained upon stimulation with Fractalkine. In fact, most of the genes that were up-regulated upon RANTES stimulation were down-regulated by Fractalkine. Specifically, RANTES binding to US28 induced expression of a number of cellular genes involved in cellular migration, while Fractalkine down-regulated many of these same genes (data not shown). These findings indicate that there are ligand-specific differences in US28 signaling that parallel the ability of either RANTES or Fractalkine to promote SMC migration.

### Ligand-Specific Signaling Mediated by US28

**Figure 2:**
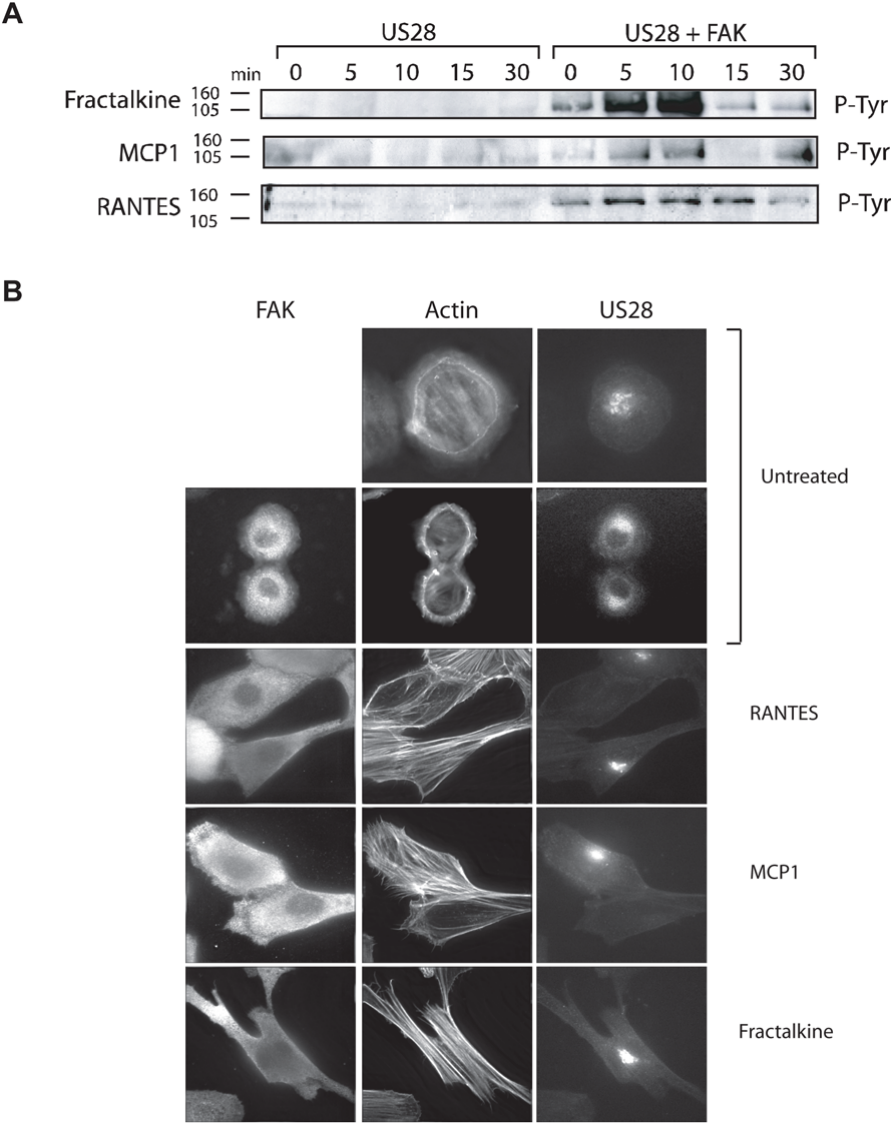
All US28 ligands are capable of activating FAK and inducing Actin Stress Fiber Formation in reconstituted FAK−/− cells. (A) FAK activation was determined by Grb2/FAK co-immunoprecipitation reactions on Ad-FAK reconstituted FAK−/− cells infected with Ad-US28 that were treated with RANTES, Fractalkine, MCP-1. Cells were harvested in modified RIPA buffer at 0 (unstimulated), 5, 10, 15, and 30 minutes post addition of ligand. Active FAK associated with Grb2 was visualized by western blotting for phospho-FAK. (B) FAK null cells infected with Ad-US28 were reconstituted with WT FAK via adenovirus transduction. RANTES, MCP-1, or Fractalkine treated cells were fixed two hours post addition of ligand. Cells were stained for actin with phalloidin (actin) and FAK using antibodies directed against the FAK-N'terminal HA-tag, and US28 using antibodies directed against the N-terminal Flag epitope present on US28. All images are 60× magnification.

To determine if the different phenotypic outcomes of RANTES or Fractalkine binding to US28 is reflected in differences at the level of signal transduction, we examined the ability each class of chemokine ligand to activate FAK through binding to US28. We have previously demonstrated that RANTES binding to US28 stimulates the activation of FAK, promoting a specific association between phosphorylated FAK and the adaptor protein Grb2. FAK is a critical mediator of focal adhesion turnover and plays important roles in cellular adhesion and motility. As such, it displays high basal activity levels in most cell types. For these experiments we developed a clean inducible signaling assay using FAK knockout mouse fibroblasts (FAK−/−) that have been reconstituted with an adenovirus vector expressing wild-type FAK concurrent with the addition of Ad-US28 [16]. To determine the ability of CC-chemokines and the CX_3_C-chemokine Fractalkine to promote US28 mediated activation of FAK and formation of active Grb2/FAK complexes, FAK−/− cells expressing US28 alone or in combination with FAK were stimulated with RANTES, MCP-1 or Fractalkine (40ng/ml) for 0 (unstimulated), 5, 10, 15 or 30 minutes. Grb2 was immunoprecipitated and active FAK associated with Grb2 visualized by western blotting for Phospho-Tyr [16]. RANTES, MCP-1 and Fractalkine all promoted US28-mediated FAK activation and formation of Grb2/FAK complexes with similar kinetics but slightly different magnitudes of activation (Figure 2A).

RANTES (CCL5)-induced signaling through US28 also promotes pronounced actin-cytoskeletal changes in multiple cell types [14]–[16]. Therefore, we also examined the ability of RANTES, MCP-1, or Fractalkine to promote actin cytoskeletal re-arrangements through US28 in FAK−/− fibroblasts. FAK−/− cells infected with adenoviruses expressing US28 and FAK were stimulated with RANTES, MCP-1, or Fractalkine (40ng/ml). Two hours post-ligand stimulation, fixed and permeabilized cells were incubated with antibodies directed against the Flag (US28) and HA (FAK) epitopes, and actin visualized by staining with Phalloidin. While RANTES, MCP-1, and Fractalkine failed to stimulate morphological changes in the absence of US28 (data not shown) each of the three ligands readily promoted actin cytoskeletal re-arrangements in US28 expressing cells (Figure 2B). Although RANTES, MCP-1 and Fractalkine differ with respect to their ability to promote SMC migration through US28, all are capable of promoting FAK activation and formation of active Grb2-FAK complexes, as well as re-organization of the actin-cytoskeleton in fibroblasts.

### Fractalkine– and RANTES–Induced FAK Activation through US28 Require Different G-proteins

**Figure 3:**
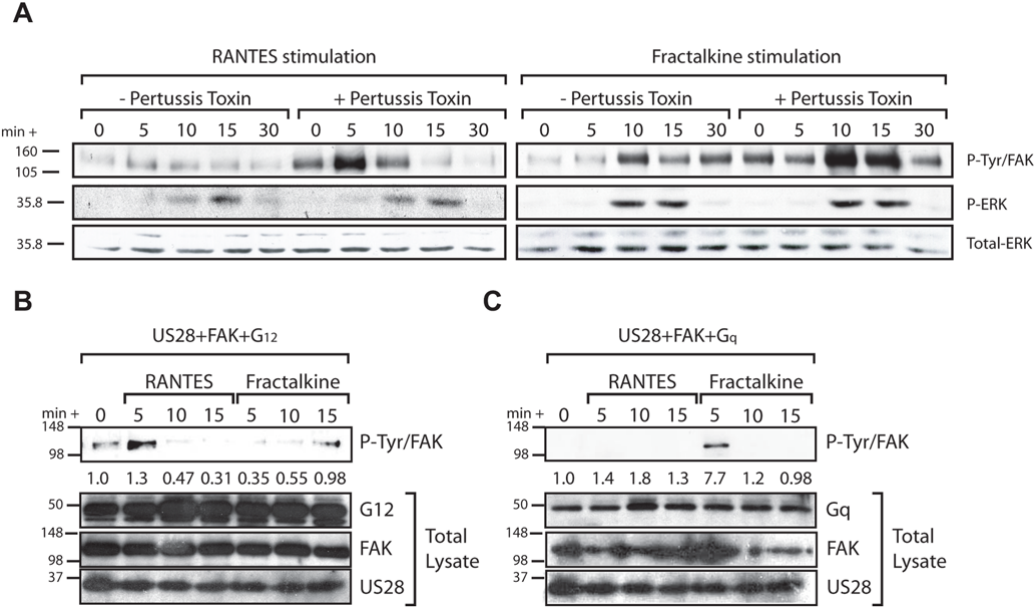
RANTES and Fractalkine activation of FAK is dependent on different G-proteins. (A) FAK activity in FAK −/− cells expressing both US28 and wt-FAK in response to either Fractalkine or RANTES and in the presence or absence of pertussis toxin was assessed by Grb2/FAK co-immunoprecipitation reactions. Cells were harvested in modified RIPA buffer at 0 (unstimulated), 5, 10, 15, and 30 minutes post addition of ligand. Active FAK associated with Grb2 was visualized by western blotting for phospho-tyrosine. (B,C) The ability of Gα12 and Gαq to enhance or abrogate RANTES and Fractalkine mediated activation of FAK through US28 was assessed by overexpressing (B) Gα12 or (C) Gαq in FAK −/− cells. FAK −/− cells infected with adenovirus expressing US28, wt-FAK and Gα12 or Gαq were stimulated with either RANTES or Fractalkine. As in (A), FAK activity was assessed by Grb2/FAK co-immunoprecipitation reactions and active FAK associated with Grb2 was visualized by western blotting for phospho-tyrosine. Western blots were quantified by densitomitry and fold FAK activation compared to unstimulated control is indicated below each lane.

Our data indicate that although CC- and CX_3_C-chemokine stimulation of US28-expressing SMC produces different migratory phenotypes, both classes of ligands are capable of activating common pro-migratory signaling cascades in US28-expressing fibroblasts. We hypothesized that the disparate phenotypes seen in US28-expressing cell types is a result of differential coupling of G-proteins to US28. To identify the G-proteins involved in RANTES and Fractalkine stimulated FAK activation through US28, Grb2-FAK co-immunoprecipitation reactions were performed on lysates from reconstituted FAK−/− cells expressing US28. Cells were pre-treated with the Gαi/o inhibitor PTX or were left untreated and then stimulated with either RANTES or Fractalkine (40ng/ml) and Grb2/FAK co-immunoprecipitations were visualized by western blotting. Pre-treatment with PTX significantly enhanced both Fractalkine and RANTES mediated activation of FAK through US28, suggesting that both ligands promote coupling to G-proteins other than Gαi/o family G-proteins to induce FAK activation (Figure 3A). Interestingly, stimulation of US28 expressing cells with either RANTES or Fractalkine led to the PTX resistant activation of ERK-1/2. Unlike US28 mediated FAK activation, which was enhanced by PTX, ERK-1/2 activation was not affected by PTX pre-treatment. Therefore, US28 mediated activation of ERK-1/2 in reconstituted FAK−/− cells is independent of Gαi/o family G-proteins, differing from PTX sensitive MCP-1 and RANTES induced ERK-2 activation by US28 observed in 293 cells [13].

We have previously determined that US28-mediated SMC migration requires the Gα12/13-dependent activation of RhoA [15]. Additionally, Fractalkine stimulation of US28 has been used as an inhibitor of Gαq/11-mediated constitutive activation of phospholipase-C (PLC) and NF-κB [10]. Since RANTES and Fractalkine induced activation of FAK through US28 is independent of Gαi/o family G-proteins, and US28 is known to signal through Gα12 to promote cellular migration in SMC, we assessed the role of Gα12 in promoting RANTES and Fractalkine mediated activation of FAK. Reconstituted FAK−/− cells infected with adenoviruses expressing US28 and wild-type Gα12 were stimulated with either RANTES or Fractalkine. FAK activation was determined using Grb2-FAK co-immunoprecipitation reactions as described above. Introduction of high levels of Gα12 had little effect on the kinetics of FAK activation by RANTES, but significantly delayed and reduced FAK activation by Fractalkine (Figure 3B). In similar assays, over-expression of Gαq abrogated RANTES-mediated FAK activation while Fractalkine mediated FAK activation was unaffected by expression of this G-protein (Figure 3C). These data are consistent with the observation that Fractalkine binding to US28 specifically decreases the constitutive activation of PLC and NF-κB via a Gαq/11 dependent mechanism. This study, combined with our previous findings, shows that US28 G-protein coupling occurs in a ligand-specific manner wherein RANTES promotes US28 coupling to Gαi/o, Gα16 and Gα12/13, while Fractalkine promotes US28 coupling to Gαq [15].

### Ligand-Specific US28 Mediated Macrophage Migration

Although Fractalkine binding to US28 fails to promote migration in SMC, we have demonstrated that Fractalkine stimulation causes cytoskeletal rearrangements and activates pro-migratory signaling pathways in fibroblasts via Gαq. Given that the endogenous complement of G-proteins differs between cell types, we hypothesized that Fractalkine binding to US28 may mediate migration of a second HCMV-susceptible cell type. Fractalkine (CX_3_CL1), is the only known CX_3_C chemokine and is unique among chemokines in that it has both membrane bound and soluble forms. Fractalkine is both a chemotactic signal for monocytes and sufficient for monocyte activation and adhesion under flow conditions [22]. HCMV infection of monocyte/macrophages is an important dissemination vehicle *in vivo* [23],[24]. We hypothesized that the capacity of US28 to bind Fractalkine with high affinity, in addition to CC-chemokine ligands, may play a role in HCMV infection of monocytes. and that, in contrast to SMC, Fractalkine stimulus may be pro-migratory in US28-expressing monocytes. We attempted these experiments in human monocytes in the context of HCMV infection. However, the presence of endogenous chemokine receptors (including RANTES-binding CCR1 and CCR5 as well as the human fractalkine receptor CX_3_CR1) and endogenous chemokine ligands in these cells made the experimental results difficult to interpret. To compensate for technical difficulties, US28 was expressed from an adenoviral vector in the context of a rat macrophage cell line. We reasoned that compared to ligands produced in human monocytes fewer endogenous rat chemokines would functionally interact with US28 and, similarly, fewer endogenously expressed rat chemokine receptors would signal productively in response to stimulation with recombinant human chemokines.

**Figure 4:**
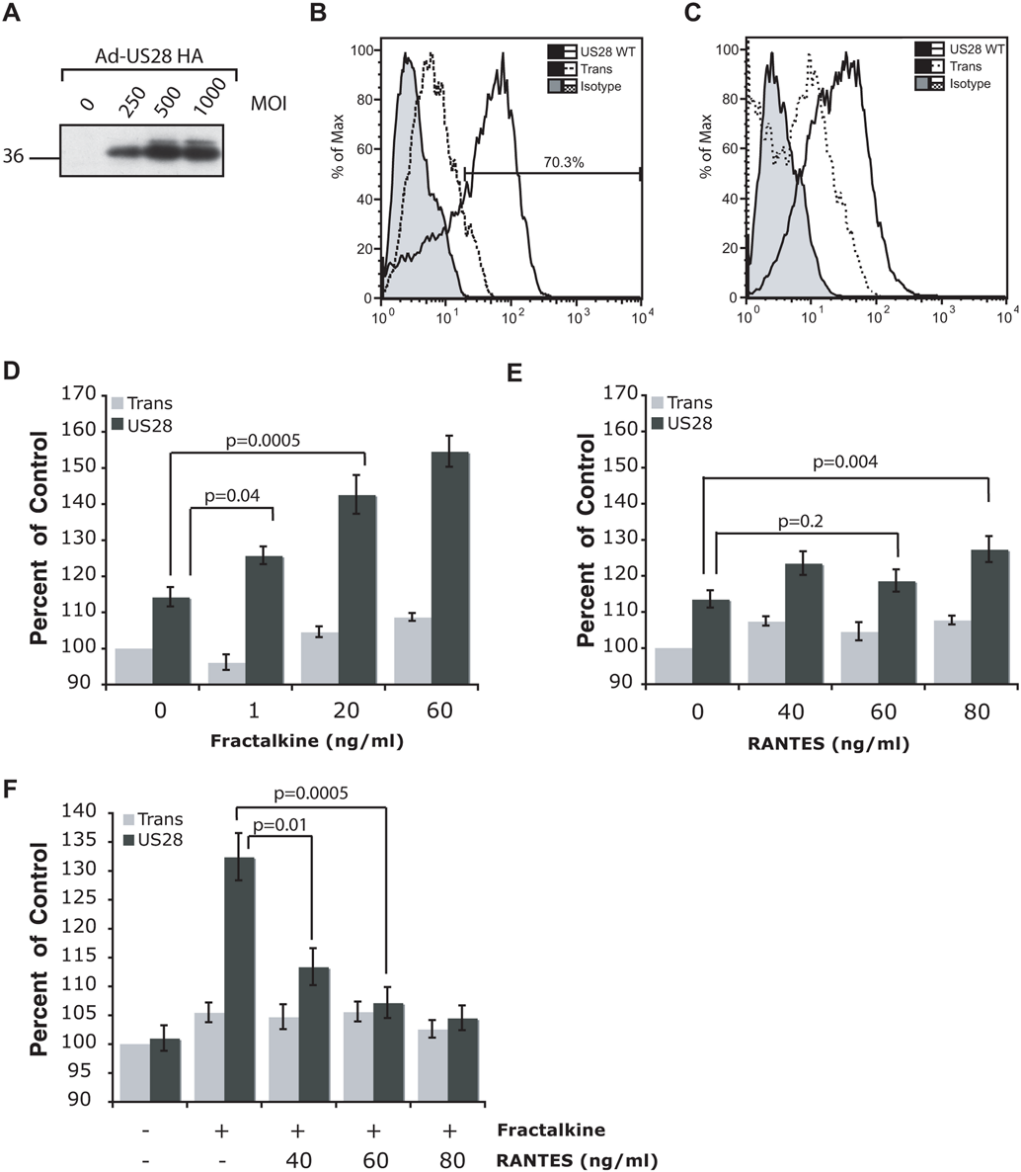
Fractalkine induces US28-mediated migration of macrophages. (A) Expression of US28 was determined via western blot analysis of total cellular lysate for the HA epitope tag. 2×10^5^ rat macrophages were infected for 72 hours with US28-HA adenovirus vector at the indicated MOI. (B) The efficiency of adenovirus transduction was determined by FACS analysis of permeablized rat macrophages infected for 72 hours at MOI 250 with US28-HA adenovirus vector. (C) Surface expression of US28 was confirmed via FACS analysis of non-permeablized rat macrophages infected for 72 hours at MOI 250 with US28-HA adenovirus vector. *In vitro* migration assays were performed on 1×10^5^ Ad-US28 and/or Ad-Trans infected rat macrophages treated with the indicated concentrations of (D) Fractalkine or (E) RANTES. For all conditions, n≥12 from four independent experiments. Percentages are calculated relative to unstimulated macrophages infected with adenovirus transactivator (Trans). (F) Competition migration assays were performed on Ad-US28 expressing macrophages treated with 40ng/ml of Fractalkine and the indicated concentrations of RANTES as a competing ligand. For all conditions, n≥12 from two independent experiments. Percentages are calculated relative to unstimulated macrophages infected with Ad-Trans.

Using a low temperature, low volume infection protocol, rat macrophages were infected with adenovirus expressing US28 at various MOI (Figure 4A). FACS analysis was used to demonstrate US28 expression in approximately 70% of permeablized macrophages stained for the HA tag (Figure 4B) and that US28 is expressed on the cell surface of adenovirus-infected macrophages. (Figure 4C). The response of US28-expressing macrophages to treatment with recombinant human RANTES and Fractalkine was assessed using a quantitative *in vitro* migration assay. In these assays, Fractalkine induced robust migration of US28-expressing macrophages (Figure 4D). Statistically significant migration was seen at very low (1ng/ml) concentrations of chemokine but not in control cells expressing only Trans. In contrast, RANTES caused weak migration of macrophages presumably due to low levels of Gα12 expressed in these cells. Only the highest dose (80ng/ml) of RANTES achieved statistical significance and this response was not titratable with increasing chemokine as seen with Fractalkine stimulation (Figure 4D and 4E). These results suggest that Fractalkine is the predominant chemotactic signal in US28-expressing macrophages. We performed chemokine competition experiments similar to those performed in SMC (Figure 1B) to determine whether RANTES and Fractalkine have any synergistic effect on US28-mediated macrophage migration. Fractalkine-dependent macrophage migration was inhibited in a dose-dependent manner by increasing concentrations of RANTES as a competing ligand (Figure 4F). These results show that, in direct contrast to results seen in SMC, RANTES is a competitive inhibitor of Fractalkine mediated macrophage migration.

**Figure 5:**
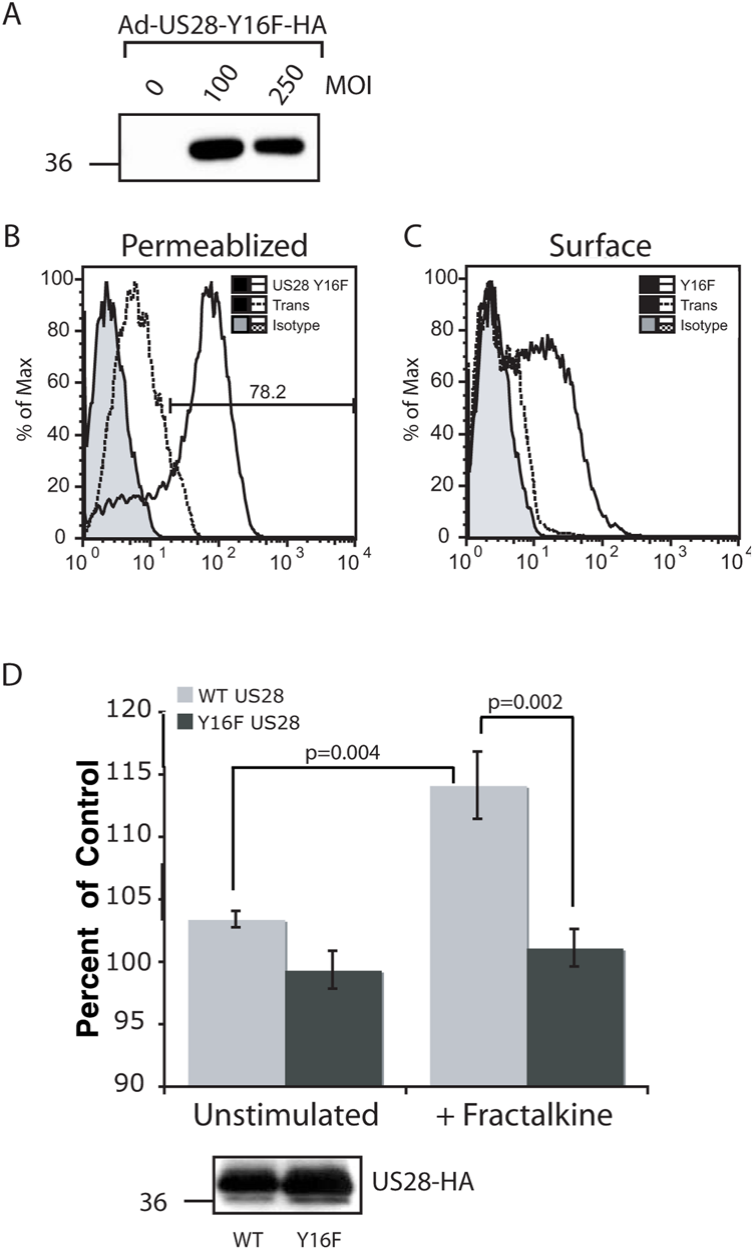
US28-mediated migration of macrophages is ligand-dependent. (A) Expression of a chemokine binding mutant US28-Y16F-HA was determined via western blot analysis of total cellular lysate for the HA epitope tag. A total of 2×10^5^ rat macrophages were infected for 72 hours with US28-Y16F-HA adenovirus vector at the indicated MOI. (B) The efficiency of adenovirus transduction was determined by FACS analysis of permeablized rat macrophages infected for 72 hours at MOI 250 with US28-Y16F-HA adenovirus vector. (C) Surface expression of US28-Y16F was confirmed via FACS analysis of rat macrophages infected for 72 hours at MOI 100 with US28-Y16F-HA adenovirus vector. (D) *In vitro* migration assays were performed on WT US28 or US28-Y16F infected rat macrophages with or without 10ng/ml Fractalkine. For all conditions, n = 8 from two independent experiments. Percentages are calculated relative to unstimulated macrophages infected with Ad-Trans. Inset is the western blot showing equal expression of US28-WT and Y16F in macrophages used for this migration assay.

To demonstrate that the US28-induced macrophage migration specifically required US28-Fractalkine interaction, we expressed the US28 mutant (Y16F), which is deficient in RANTES and Fractalkine binding [12]. US28-Y16F is efficiently expressed in adenovirus-infected macrophages (Figure 5A) and is present on the cell surface (Figure 5B). Macrophages expressing Y16F mutant did not migrate in response to Fractalkine (Figure 5C). Taken together these results demonstrate that US28-expressing macrophages respond to stimulus with recombinant human chemokine in a ligand-specific manner. Furthermore, in contrast to the CC-chemokine mediated migration phenotype in SMC, Fractalkine binding to US28 produces robust migration in macrophages. These are the first data to demonstrate a specific cellular phenotype mediated by US28 binding to Fractalkine and the first example of ligand-specific chemotaxis mediated by a multiple chemokine family binding receptor.

## Discussion

In the current report, by examining the functional responses, signaling characteristics, and transcriptional profiles induced by US28 upon binding a diversity of ligands, we demonstrate that not only is US28-signaling ligand and cell-type dependent but also ligand and cell type-specific. While RANTES stimulation of US28 causes robust SMC migration, Fractalkine provides an anti-migratory signal in these cells. Similarly, RANTES but not Fractalkine increases transcription of genes involved in SMC migration. In contrast, Fractalkine but not RANTES provides a strong chemotactic stimulus for US28-expressing macrophages, and RANTES is able to competitively inhibit Fractalkine-mediated macrophage migration. Interestingly, while these ligands display differential signaling characteristics with respect to cellular migration, they both are capable of activating FAK and producing actin cytoskeletal rearrangements in fibroblasts. Importantly, we demonstrate that these phenotypic differences can be attributed to RANTES and Fractalkine causing differential G-protein coupling to US28. Fractalkine induced-US28 signaling occurs in a Gαq-dependent manner and is abrogated in the presence of Gα12 but not by PTX. However, RANTES induced migration and signal transduction occurrs in a Gα12 dependent manner and is blocked by overexpression of Gαq. Ultimately, our findings indicate that US28 binding to RANTES or Fractalkine results in differential G-protein coupling/activation leading to unique functional consequences.

While most chemokine receptors bind a limited subset of chemokines from a single chemokine subfamily, there are three examples of chemokine receptors that bind chemokines from multiple subfamilies: the DARC-receptor, Orf74 of HHV-8, and US28 [5]–[7],[11]. To date DARC, which binds both CC- and CXC-chemokines (CCL2, CCL5, CXCL1, and CXCL8), is the only true chemokine sink because this receptor binds and internalizes these ligands without inducing signaling events. Orf74 has also been demonstrated to bind both CC- and CXC-chemokines; however, there is a significant difference in the affinity of individual ligands for this receptor. Despite being referred to as an IL-8 receptor, Orf74 has greater affinity for GRO peptides (αβγ) than for IL-8 [6]. In competition binding assays with IL-8, Orf74 binding to the CC-chemokines MIP-1α, MIP-1β, MCP-1, and RANTES was virtually undetectable, while MCP-3 and aminooxypentane (AOP)-RANTES display affinities in the 200nm range. Interestingly, the structurally distinct, non-ELR containing CXC-chemokines IP-10 and SDF-1α can displace IL-8 binding, and function as efficient inverse agonists of Orf74 signaling at nanomolar concentrations [6]. Although Orf74 binds to chemokines from multiple chemokine subfamilies, Orf74 signaling only occurs in the presence of ELR, and pro-inflammatory/angiogenic chemokines, whereas the angiostatic non-ELR CXC-chemokines function as efficient inverse agonists. Unlike Orf74, US28 binds multiple ligands from different chemokine subfamilies with near equal affinity [9],[11], and as we demonstrate in the current report, these distinct ligands promote cellular activation upon binding US28. Therefore, to date, US28 is the only chemokine receptor capable of signaling upon binding ligands from multiple chemokine subfamilies.

We have demonstrated that both MCP-1 and RANTES promote US28-mediated SMC migration [14]. While Fractalkine is a known modulator of US28-induced constitutive signaling activity [18],[19], we have shown that Fractalkine does not promote US28-mediated SMC migration and actually inhibited RANTES mediated SMC migration. In accordance with these ligand-specific functional responses, microarray analysis of US28-expressing SMC stimulated with either RANTES or Fractalkine revealed profound differences at the level of gene induction. In the context of CMV-infection of SMC, the ability of US28 to adhere to mobilized Fractalkine, coupled with our finding that this chemokine reverses transcriptional activation required for cellular migration in SMC, suggests that Fractalkine may arrest US28-induced SMC migration and promote the subsequent adhesion of US28 expressing SMC to the vascular endothelium. The migration of HCMV infected and US28 expressing SMC from the vessel media to inflammatory sites in the vessel intima and the subsequent adhesion and accumulation of SMC in the vessel intima may have important implications in the dissemination and *in vivo* pathogenesis of HCMV, as well as in the exacerbation of vascular disease.

In this study, we also demonstrate that Fractalkine causes robust migration of US28-expressing macrophages, which is the first known cellular phenotype associated with Fractalkine binding to US28. This finding indicates that, in addition to being ligand-dependent and ligand-specific, the function of US28 signaling is also cell type-specific. Our finding that Fractalkine causes migration of US28-expressing macrophages suggests a further role for US28 in the development of vascular disease. US28 has been shown to be expressed in HCMV-infected peripheral blood mononuclear cells [25]. Foam cells found in atherosclerotic lesions originate as circulating monocytes and chemokines play an important role in the deposition of monocytes in lesions [26]. In particular, Fractalkine expression is known to be important for the development of atherosclerosis in mouse models of heart disease via recruitment of macrophages into atherosclerotic plaques [27],[28]. Expression of membrane-bound Fractalkine can be induced on endothelial cells by numerous cytokines including IFN-γ, TNF-α and IL-1, resulting in the recruitment of inflammatory cells and contributing to chronic inflammatory vascular diseases such as atheroscleorosis, restenosis following angioplasty and transplant vascular sclerosis [29]. Unlike other chemokines which are secreted as soluble molecules that must associate with proteoglycans and other components of the extracellular matrix to establish chemokine gradients [30], Fractalkine is generated as a membrane bound ligand with the chemokine domain presented at the top of the cell-bound mucin-like stalk [29],[31]. In many instances this ligand is more effective than other ligands in promoting leukocyte activation and migration. Our current findings suggest a secondary mechanism for US28 in CMV-mediated vascular pathology by which circulating CMV-positive monocytes infiltrate atherosclerotic plaques mediated by Fractalkine binding to US28.

We demonstrate for the first time that Fractalkine is a potent agonist capable of inducing cellular migration in macrophages and activation of signaling pathways upon binding US28. Prior to this study, Fractalkine had been employed as a modulator of US28-mediated constitutive signaling activity. Some of the signaling pathways activated by Fractalkine were similar to those activated by the CC-chemokines. For example, RANTES, MCP-1, and Fractalkine all display similar abilities to induce ERK-1/2, actin cytoskeletal rearrangements and formation FAK-Grb2 complexes in fibroblasts. Pre-treatment with PTX enhanced Fractalkine mediated FAK activation through US28, which indicated that Fractalkine promoted US28 coupling to G-proteins other than Gai/o. Expression of Gα12 delayed and reduced FAK activity via Fractalkine signaling through US28 but had no effect on RANTES/US28 activation of FAK. Importantly, overexpression of Gαq blocked RANTES signaling to FAK but had no effect on Fractalkine-mediated FAK activation. In a number of different activation scenarios FAK is a known point of signaling convergence and has been demonstrated to be phosphorylated in response to Gαq/11, Gαi/o, and Gα12/13 coupled receptors in various cell types and signaling environments [32]–[35]. In one study, lysophosphatidic acid (LPA) signaling stimulated both membrane association and autophosphorylation of FAK but these two effects were separable and mediated by different G-alpha subunits (Gαi1 and Gα12/13, respectively) presumably via signaling from two different LPA receptors [33]. Importantly, in a receptor-decoupled system of constitutively active G-alpha subunits, significant FAK phosphorylation can be observed via signaling through Gαq, Gα12 and Gα13 [35]. These results are consistent with our observations that both RANTES and Fractalkine binding to US28 can activate FAK via different signaling cascades mediated by different G-proteins. Our results suggest that overexpression of off-target G-proteins inhibit signaling from a particular ligand via competition with the G-proteins that would normally promote signaling from the ligand-bound activated receptor. Therefore, in these experiments overexpression of Gα12 may act as a dominant inhibitory molecule that prevents Gαq-receptor interactions, which would normally activate FAK following Fractalkine coupling to US28. Overexpression of Gαq prevents Gα12 coupling to the RANTES-bound activated form of US28 thereby abrogating the downstream signaling to FAK. Therefore, RANTES stimulates varying signaling pathways through different G-proteins in SMC (Gα12-dependent) and fibroblasts (Gαi/o independent). Fractalkine signals from US28 via coupling of Gαq in fibroblasts, SMC and macrophages. Together these findings demonstrate that not only is US28 signaling ligand-dependent and ligand-specific, it utilizes differential G-protein coupling to produce cell-type specific signaling and differential phenotypic responses.

In this report, we demonstrate that similar to RANTES and MCP-1, Fractalkine is a potent US28 agonist that promotes migration in macrophages, robust signaling through FAK and ERK1/2 and induces actin cytoskeletal rearrangements in fibroblasts. Unlike RANTES and MCP-1, Fractalkine fails to induce SMC migration, or increase expression of cellular genes involved in motility and signaling in SMC, thus demonstrating that US28 signaling is ligand-specific and cell type-specific. In addition, the US28 ligand-specific and cell-type dependent activation of differential signaling pathways suggest that this chemokine receptor has the capacity to couple to different G-proteins depending upon the ligand bound and the cellular G-protein environment. Therefore, US28 binds to a diversity of chemokines, which promote US28 coupling to multiple G-proteins, eliciting functional signaling through these various G-proteins. HCMV encounters and infects a multitude of distinct cell types *in vivo* including fibroblasts, monocyte/macrophages, endothelial cells and SMC. These cell types differ substantially with respect to the G-proteins that they express. The ability of US28 to respond to multiple signaling environments and couple to multiple G-proteins may have important implications in the persistence and pathogenesis of HCMV in these different cell-types.

## Materials and Methods

### Cell Lines

The life-extended human pulmonary artery smooth muscle cell line, PAT1 [15] were maintained in Medium 199 supplemented with 20% fetal calf serum (FCS) and penicillin-streptomycin-L-glutamine (PSG; Gibco). For migration and microarray experiments, PAT1 cells were utilized between passage 5 and 30 post-telomerization. Primary F344 rat smooth muscle cells (RSMC) were maintained in Dulbecco's modified Eagle's Medium (DMEM) with 10% FCS and PSG. RSMC were used between passage 5 and 20. NR8383 rat alveolar macrophages were maintained in RPMI with 10% FCS and PSG. Mouse FAK−/− fibroblasts were maintained on gelatin coated culture dishes in DMEM supplemented with 10% FCS, PSG, non-essential amino acids (Cellgro), and G418 (Sigma; 500 µg/ml) as previously described [36],[37]. FAK−/− cells used in experiments were between passage 5 and 15.

### Reagents

Recombinant human RANTES, MCP-1, and Fractalkine were purchased from R&D Systems. Anti-Grb2 (C-7), anti-phosphotyrosine (PY99), anti-Gα12 (S-20), anti-Gαq (E-17) and anti-HA (F-7) antibodies were purchased from Santa Cruz Biotechnology. Phospho-specific ERK-1/2 (Thr202/Tyr204) and total ERK-1/2 antibodies were from Cell Signaling Technologies. Anti-M2-Flag antibody (F-3165) was purchased from Sigma. Secondary anti-mouse and anti-rabbit horseradish peroxidase (HRP)-conjugated antibodies (NA934V and NA931V) were purchased from Amersham.

### Adenovirus Construction

Adenoviruses expressing Gα12 Gαq, WT-FAK, US28-Flag, and US28-HA were previously described [14]–[16]. Adenovirus vectors expressing US28-Y16F-HA were constructed by mutagenesis of the US28-HA construct in pAdTet7. This vector contains the tet-responsive enhancer within a minimal CMV promoter followed by the SV40 late poly(A) cassette, adenovirus E1A, and a single loxP site to increase recombination frequency. Complementary 30bp primers containing coding sequence for amino-acids 2–25 of US28-HA and including a phenylalanine codon in place of the tyrosine at position 16 (5′-ACGACGGAGTTTGACTTCGACGATGAAGCG-3′ and 5′-CGCTTCATCGTCGAAGTCAAACTCCGTCGT-3′) were used to PCR amplify mutated vector using Pfu Turbo DNA Polymerase (Stratagene). Non-mutated methylated parental DNA was digested using *DpnI* and mutated plasmid was propagated in DH5α. Recombinant adenoviruses were produced by pAdTet7 US28-Y16F-HA construct co-transfection of 293 cells expressing the Cre-recombinase with adenovirus DNA (Ad5-ψ5) that contains an E1A/E3-deleted adenovirus genome [38]. Recombinant adenoviruses were expanded on 293-Cre cells and the bulk stocks were titered on 293 cells by limiting dilution. Gene expression was driven by co-infection with Ad-Trans expressing the Tet-off transactivator as previously described [14].

### Flow Cytometry

To monitor surface expression of recombinant proteins and total adenovirus transduction, adenovirus-infected cells were fixed in 2% PFA for 15min, washed 2× with PBS, blocked for 15min on ice in Fc Block (PBS+20%Normal goat serum (NGS)+0.1% sodium azide). To determine the rate of adenovirus transduction, cells were permeablized with PBS containing 0.2% Saponin and 0.02%NGS for 15min on ice. For both cell surface and intracellular staining assays the cells were incubated for 30min with either mouse IgG2b isotype control or primary αHA antibody diluted 1∶200 in FACS wash buffer (PBS+1% NGS+0.01% sodium azide +/− 0.2% saponin as appropriate) on ice and washed 2× with FACS wash buffer. Primary antibody staining was detected with anti-mouse Alexa-Fluor597 antibody diluted 1∶1000 in FACS wash. After 20 min incubation with secondary antibody on ice cells were washed as above and surface expression was quantified using flow cytometry (FACS Calibur, BD Biosystems). Data analysis was performed using FlowJo software v8.8 (Treestar Inc.).

### Immunocytochemistry

FAK**^−/−^** fibroblasts were grown in 0.1% gelatin coated 4-well chamber slides (Nalge-Nunc). US28 and/or FAK was expressed using the adenovirus vectors described above and were left untreated or were treated with MCP-1, RANTES or Fractalkine (20ng/ml) for 2 hrs. The cells were washed in PBS and fixed in phosphate buffered 2% paraformaldehyde (PFA) for 15 minutes at r.t., then permeabilized and blocked with 0.2% Saponin+0.02% BSA in PBS for 15min at r.t. Thereafter, the cells were incubated with antibodies against US28-Flag epitope or FAK-HA epitope in a 1∶200 dilution for 1 hr at room temperature. Cells were washed three times in blocking buffer and binding of the primary antibody was detected with a fluorescein isothiocyanate-tetramethyl (FITC) conjugated goat anti-mouse or rhodamine conjugated goat anti-rabbit antibody for 1 hr at room temperature. At this time the cells were also stained for actin using Phalloidin (Molecular Probes, Eugene, OR) to monitor alterations in cellular actin cytoskeleton induced by US28 and FAK. Fluorescence positive cells were visualized on an inverted Applied Precision Deltavision™ deconvolution microscope.

### Immunoprecipitation Reactions

FAK−/− cells were plated in 10cm culture dishes and serum starved for 6 hrs upon achieving 50% confluence. The cells were co-infected with Ad-Trans and/or Ad-US28 and/or Ad-FAK WT at MOI 50. After 16 hrs the cells were stimulated with RANTES (40ng/ml), Fractalkine (40ng/ml), or MCP-1 (40ng/ml) and then harvested at times 0 (unstimulated), 5, 10, 15, and 30 minutes post ligand addition. Cells were lysed in modified RIPA buffer containing 1% Triton X-100, 1% sodium deoxycholate, and 0.1% SDS and total Grb2 was immunoprecipitated and samples analyzed by western blotting using antibodies directed against phospho-Tyr [16]. Co-precipitation of FAK-HA was demonstrated by stripping the blots in buffer containing 0.1M Tris pH 6.8, 1% SDS, and 1% 2-mercaptoethanol and staining using antibodies directed against HA. Prior to immune-complex reactions, a total of 50 µl of cellular lysate was assayed by SDS-PAGE/western blotting for the presence of input US28 and FAK using antibodies directed against the HA-epitope present on both recombinant proteins.

### SMC Migration Assay

SMC migration assays were performed as previously described [14]. Briefly, 4×10^4^ primary rat SMCs were added to each upper well of a transwell (12 mm diameter, 3.0 µm pore size, Costar Corning, Cambridge, MA). Cells were serum starved for 16 hrs, and then infected with Ad-Trans only or Ad-Trans and Ad-US28-HA at MOI 200. After 4 hrs, the inserts were washed and transferred to fresh 12-well plates with chemotactic stimulus. Cells migrating to the lower chamber were quantified at 48–72 hrs p.i. via fluorescence using CyQuant (Invitrogen) and read on a Molecular Devices Flexstation® II fluorescence plate reader. Migration was determined from 4–6 independent wells per assay per condition. Mean and standard deviation were calculated. Percent of control values were generated by comparing chemokine stimulated US28-expressing cells to unstimulated control cells (Trans-only) and compared using Student's *t* test. *P* values<0.05 were considered statistically significant.

### Macrophage Migration Assay

NR8383 macrophages were co-infected with Ad-Trans and Ad-US28 WT or Ad-US28-Y16F at MOI 100. Macrophages were incubated with adenovirus in 200 µl total volume for 30min at room temperature, diluted into 10ml complete RPMI and incubated at 37°C. At 72 hrs post-infection, 1×10^5^ infected macrophages were added to the top well of a chemotaxis chamber (96-well Millipore Multiscreen, 3.0 µm pore size) with Fractalkine and/or human RANTES in the bottom chamber. Chemotaxis was allowed to proceed for 1 hr at 37°C. Top chambers were discarded and migrated cells in the bottom chamber were quantified via fluorescence using CyQuant (Invitrogen) and read on a Molecular Devices Flexstation II fluorescence plate reader. Migration was determined from 4–6 independent wells per assay per condition. Mean and standard deviation were calculated. Percent of control values were generated by comparing chemokine stimulated US28-expressing cells to unstimulated control cells (Trans-only) and compared using Student's *t* test. *P* values<0.05 were considered statistically significant. Recombinant protein levels were monitored by western blotting and flow cytometry staining for total and surface expression and equalized by adjusting the adenoviral vector MOI accordingly.
